# Access Options for Transcatheter Aortic Valve Replacement

**DOI:** 10.3390/jcm14051651

**Published:** 2025-02-28

**Authors:** Jeffrey Chidester, Teodora Donisan, Parth V. Desai, Sukriti Banthiya, Ahmed Zaghloul, Michael E. Jessen, Ki Park, Weiyi Tan, Shirling Tsai, Lynn Huffman, Anthony A. Bavry, Dharam J. Kumbhani, Amit Goyal

**Affiliations:** 1 Division of Cardiovascular Medicine, University of Virginia, Charlottesville, VA 22903, USA; 2 Mayo Clinic, Rochester, MN 55905, USA; 3 CardioNerds, Baltimore, MD 21209, USA; 4 UT Southwestern Medical Center, Dallas, TX 75390, USA; 5 Ascension Providence Hospital, Michigan State University, Southfield, MI 48075, USA; 6 Veterans Affairs North Texas Health Care System, Dallas, TX 75216, USA

**Keywords:** arterial access, transcatheter aortic valve replacement, transcaval, transcarotid, transapical, transaortic, transfemoral, transaxillary

## Abstract

Transcatheter aortic valve replacement (TAVR) was introduced in 2002 and has become integral in the management of aortic stenosis. As an alternative to surgical aortic valve replacement, it relies heavily on safe access to the aortic annulus for implantation of a valve prosthesis. Throughout its development and in current practice, the transfemoral (TF) arterial route for retrograde valve delivery has been the primary approach. However, this route is not appropriate for all patients, which has led to the development of multiple alternate access options. This review discusses the development of access for TAVR, followed by a thorough discussion of TF access. The commercially available products, preprocedural planning, closure techniques, and procedural complications are all discussed. We also describe the various alternate access routes with particular emphasis on the most recently developed route, transcaval access (TCv), with focus on procedural indications, technical considerations, and comparative outcomes. As TAVR technology, indications, and availability all expand, the knowledge and implementation of safe access are of utmost importance.

## 1. Introduction

Severe aortic stenosis is a progressive condition without effective medical therapy that traditionally requires surgical aortic valve replacement [1]. The development of transcatheter aortic valve replacement (TAVR) has revolutionized the management of this disease. Since the first-in-human case was performed in 2002, iterative technological advancements, an expanding evidence base, and increased operator experience have all led to a wider eligible population undergoing TAVR [2]. Additionally, ongoing trials may expand the procedural indications to include moderate aortic stenosis in certain situations and aortic regurgitation. In 2015 TAVR surpassed isolated surgical aortic valve replacement (SAVR) in the United States, and in 2018 it surpassed total SAVR (includes combined operations) [3]. The key benefits of the transcatheter approach are avoiding sternotomy and cardiopulmonary bypass. This allows for quicker recovery and avoids the complications associated with surgical aortic valve replacement. As such, safe arterial access to perform the procedure effectively is paramount. The high prevalence of vascular disease in these patients, who are often of advanced age and with multiple comorbidities, has led to the development of a variety of access techniques to facilitate device delivery through the panoply of challenging vascular access. This review discusses the development and features of established vascular access techniques for performing TAVR with particular emphasis on the transfemoral (TF) and transcaval (TCv) routes.

## 2. Evolution of TAVR Access

### 2.1. Early Development

The TAVR procedure entails the deployment of a bioprosthetic valve in the aortic annulus. While chiefly performed for aortic stenosis, the initial intravascular prostheses conceived for treating aortic valve disease were developed to address aortic regurgitation. These parachute-like and balloon devices were designed to be positioned in the ascending aorta, allowing for antegrade flow while preventing retrograde flow either by the nature of their design or by augmentation at appropriate points in the cardiac cycle. They were inserted via the carotid artery; however, all required an external control system without provisions for a durable implant. As such, these never became viable for managing aortic regurgitation. Further development led to the first described in vivo deployment of a bioprosthetic aortic valve in pigs. A porcine aortic valve was sutured to a wireframe and compressed; however, it required a 41 F access obtained via a midline laparotomy. This large access requirement limited the clinical translation of this technology [4].

### 2.2. First In-Human Case

In 2002, Dr. Alain Cribier published the first case of transcatheter aortic valve implantation in a human, a 57-year-old man with severe aortic stenosis and acute decompensated heart failure who had disease refractory to balloon valvuloplasty. Due to the patient’s bilateral femoral arterial disease, both the initial balloon valvuloplasty and subsequent transcatheter valve implantation were performed antegrade via a transseptal approach, with percutaneous 24 F transfemoral venous access. Despite short-term improvement and excellent valve performance, the patient ultimately succumbed to progressive right lower limb ischemia and complications related to amputation [2].

### 2.3. Retrograde Development

Three years after Dr. Cribier’s initial case, Dr. David Paniagua published the first report of retrograde aortic valve implantation via percutaneous femoral arterial access. The self-named “Paniagua valve” was first tested in sheep and calf subjects with either a 16 F or 11 F sheath used to implant the valves via surgically exposed carotid or femoral arterial access. The first human to receive this valve was a 62-year-old man with severe vascular disease, severe aortic stenosis, acute decompensated heart failure, renal failure, and pulmonary hypertension. The femoral artery was surgically exposed, and a 16 F sheath was advanced to the abdominal aorta for aortic valve device implantation. The valve was successfully deployed, and the patient had dramatic short-term improvement; however, he also succumbed to his underlying illness [5]. This and Dr. Cribier’s initial case both demonstrate the impact of comorbid vascular disease and delivery system size on the method of access. Access considerations continue to inform the development and current landscape of vascular access for TAVR.

### 2.4. Progression of Alternate Access Development

**Figure 1:**
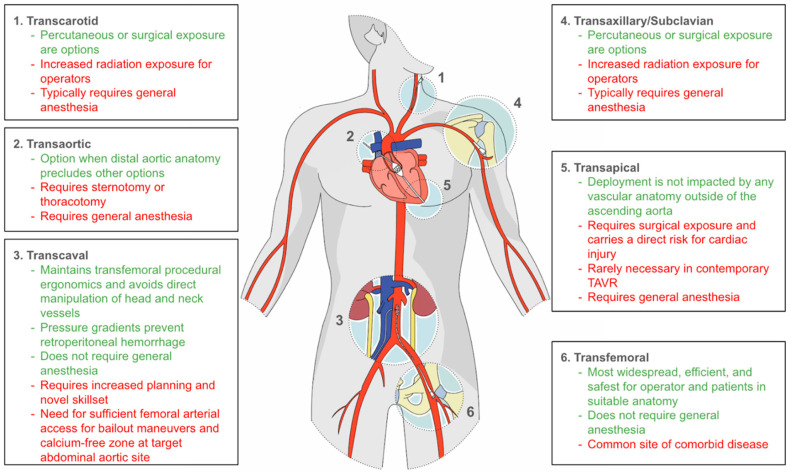
TAVR Access Sites with Site-specific Considerations. Adapted from [11].

**Figure 2:**
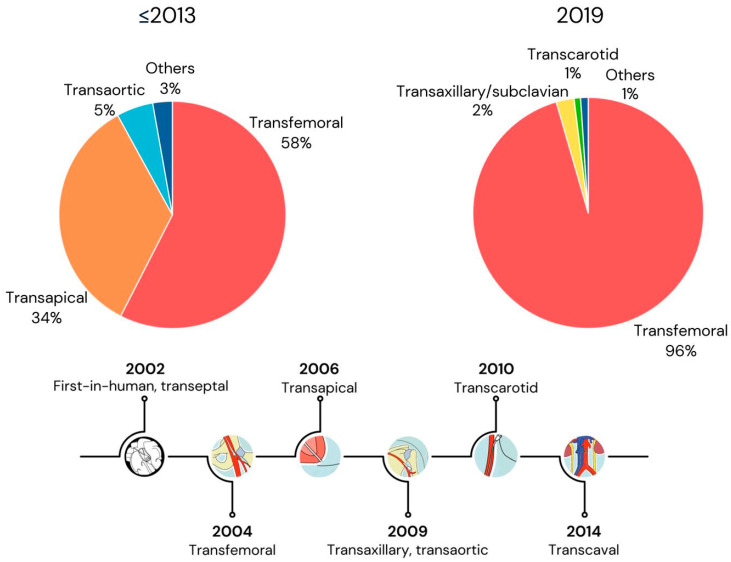
Chronologic Development and Frequency of TAVR Access Sites. Adapted from [3].

Since the introduction of TAVR, most procedures have been performed through the retrograde percutaneous transfemoral (TF) arterial route [3]. For those with insufficient femoral and/or challenging aortic anatomy, alternative approaches have been developed. These include transapical (TA), first described in 2006; transaxillary/subclavian (TAx), first described in 2009; transaortic (TAo), first described in 2009; transcarotid (TC), first described in 2010; and transcaval (TCv), first described in 2014 [6,7,8,9,10]. Transfemoral access (TF, hereafter referring to transfemoral arterial access) continues to be the predominant method of performing TAVR, with an increasing proportion of procedures performed via this route over time. Before 2013, 57% of procedures were transfemoral, with TA (34%) and TAo (5%) being the most commonly used alternative access methods. This has changed dramatically, with 2019 data demonstrating 96% TF access, with transcarotid being the most commonly utilized alternative access site (2.1%). In contrast to cases performed before 2013, TA and TAo access were utilized in <0.5% of cases in 2019 (Figure 1 and Figure 2) [3].

## 3. Transfemoral Access

Transfemoral arterial access (TF) allows for retrograde access to the aortic valve for prosthesis implant. Access is typically performed percutaneously; however, surgical cutdown can be performed in the setting of diseased or anatomically complex common femoral arteries. Surgical cutdown allows for direct visualization and repair of the artery. Open access often requires general anesthesia, and while safe, it can increase hospital length of stay. Techniques developed for safe large-bore percutaneous vascular access and closure for transfemoral coronary procedures and temporary mechanical circulatory support have been adopted for TF-TAVR. Additionally, the profile of newer generations of TAVR delivery devices has decreased, improving procedural safety [12]. Briefly, arterial access is obtained in the common femoral artery proximal to the bifurcation of the superficial femoral artery and the profunda femoris artery and distal to the inferior epigastric artery. This site helps prevent retroperitoneal hemorrhage and enables both effective manual compression and safe use of vascular closure devices. The integration of computed tomography, intravascular ultrasound, and fluoroscopy allows for pre-procedural identification of appropriate anatomy and intraprocedural guidance to facilitate large-bore femoral arterial access. The information obtained from this multimodality imaging is then integrated with the characteristics of available valve delivery systems for the safe performance of TF TAVR.

### 3.1. Current Commercial Devices

There are currently three commercially available TAVR valves with their unique delivery systems. Considerations should be made regarding the delivery of each valve and its suitability for an individual patient’s anatomy or access route; valve selection is outside of the scope of this review. Basic considerations of the three commercially available valve platforms are discussed below.

#### 3.1.1. Edwards Commander/Sapien System

The Commander delivery system is used to deliver the Sapien balloon-expandable valve (Edwards Lifesciences, Irvine, CA, USA). The valve comes in four sizes: the smaller three, which require a ‘14 F equivalent’ (outer diameter 6.0 mm) sheath, and the larger one, which requires a ‘16 F equivalent’ (outer diameter 6.7 mm) sheath. The recommended minimum vessel diameters are 5.5 mm and 6.0 mm for the small and large sheaths, respectively. The Edwards sheath has a seam that is oriented dorsally to avoid injury. This allows for transient expansion of the sheath and facilitates the passage of the unmounted valve and its delivery system while maintaining a lower-profile arteriotomy [13].

#### 3.1.2. Medtronic Evolut System

The Evolut system (Medtronic, Dublin, Ireland) is a self-expanding valve that is implanted via an in-line sheath that is inserted after left ventricular access is obtained. The valve comes in four sizes: the smaller three, which require a ‘14 F equivalent’ (outer diameter 6.0 mm) sheath, and the larger one, which requires an ‘18 F equivalent’ (outer diameter 7.33 mm) access. The recommended minimum vessel diameters are 5.0 and 6.0 mm for the small and large sheaths, respectively [14].

#### 3.1.3. Abbott Navitor System

The Navitor system (Abbott, Lake County, IL, USA) is a self-expanding valve implanted via an in-line sheath inserted after left ventricular access is obtained. The valve comes in five sizes. The valves are delivered through two possible sheaths: a “Small”, which is ‘14 F equivalent’ (outer diameter 6.0 mm), and a “Large”, which is ‘15 F equivalent’ (outer diameter 6.3 mm). The recommended minimum vessel diameters are 5.0 and 5.5 mm for the small and large sheaths, respectively [15].

Iterative modifications of these valve systems, as well as the commercial introduction of new valve platforms, will likely impact access considerations in the future. The Edwards and Medtronic valve platforms have undergone a continued reduction in sheath size throughout their commercial availability. It is important to note that the minimum required diameters may be larger in the presence of prior stents or concentric calcification. 

### 3.2. CT Planning

Computed tomography (CT) has become the standard of care in TAVR planning. It allows for both the assessment of access sites as well as the determination of the appropriate prosthesis size and design for the patient’s anatomy. CT represents an advancement from iliofemoral angiography and has facilitated improved assessment and procedural outcomes [16,17,18]. A consensus document published by the Society of Cardiovascular Computed Tomography in 2019 covers the specifics of this technology in facilitating TAVR [16]. In contrast to imaging performed for assessing cardiac structures, CT for aorto-iliofemoral vasculature does not require ECG gating; however, like cardiac structures, vascular assessment is improved with the use of contrast. This modality, with available three-dimensional reconstruction software, allows for the assessment of vessel size, calcification, and tortuosity, as well as the location of the common femoral artery bifurcation. Abnormal aortic anatomy (such as aneurysms or chronic dissections) that may preclude TF access can also be identified. In addition, the images obtained from a complete contrast-enhanced CT of the chest, abdomen, and pelvis to determine the feasibility of TF TAVR are also informative in alternate access planning in the event that TF is not an appropriate option.

#### 3.2.1. Vessel Size

CT imaging allows for longitudinal, discrete assessment of vessel size, typically from the level of the mid-thigh to the aortic annulus. Vascular anatomy, particularly the minimal luminal diameter, helps determine whether the necessary sheath size can be safely inserted for valve delivery. This can be measured from the level of the common femoral artery to the distal aorta, with the minimum diameter (either due to small anatomy or plaque) reported. Traditionally, a sheath/minimum diameter ratio of less than or equal to 1.05 is felt to allow safe TF access [16]. The use of more liberal thresholds and other techniques have been described to identify what ratio is appropriate [19].

#### 3.2.2. Calcification

Peripheral arterial disease is common in patients undergoing TAVR [20]. Comprehensive CT vascular assessment will note the presence, distribution, and location of calcified lesions due to the role these play in impeding device delivery and as a mechanism of complications [16]. This is often described in semi-quantitative categories as no calcification, mild (< 90° of total circumferential arc), moderate (90–180° of total circumferential arc), marked (180–270° of total circumferential arc), and severe calcification (>270° of total circumferential arc). It is also important to consider the length of calcified segments, as longer segments will limit the distensibility and mobility of the vessel to a greater extent.

#### 3.2.3. Tortuosity

Tortuosity refers to the degree of angulation in a vessel, particularly when there are serial segments with alternating extreme angulation. In many situations, the flexibility of the delivery sheaths and the support that can be gained from increasingly stiff delivery wires can overcome tortuosity. Nevertheless, extreme tortuosity can impede delivery and predispose to complications. This is often a subjective assessment; however, there have been objective methods defined, including reporting the largest single angle in the vessel, a sum of all angles, and looking at a ratio of the true vessel length to the ideal vessel length (the straight-line distance within the body, which it covers) [21]. Tortuosity may be of higher concern when tortuous segments are calcified, limiting their mobility.

#### 3.2.4. Predictor of Adverse Events

The identification of high-risk TF anatomic features should trigger consideration for alternative access options as well as evaluation for the feasibility of endovascular options to facilitate TF delivery. The appropriateness of TF access is not binary, but a range spanning healthy to prohibitive. Still, even optimal TF anatomy may succumb to vascular access complications. Several methods have been developed for risk stratification based on the CT. The individual components described above all confer some risk for access-related complications. The HOSTILE score utilizes seven CT-derived variables: number of iliofemoral lesions, presence of obstruction in any segment, iliac disease involving the aortic bifurcation, lesions located in a tortuous segment, >180° calcified lesions, lesion length > 100 mm, minimal luminal diameter < 5 mm. This score was predictive of non-puncture site vascular complications and may represent an effective tool for identifying patients at higher risk [22,23].

### 3.3. Ultrasound Guidance

The use of ultrasound guidance for all medical procedures has increased, as it has for TF arterial access in the performance of coronary angiography. Procedural ultrasound is increasingly available and does not require contrast or radiation. Given the large sheath sizes utilized in TAVR and the reliance on vascular closure devices (which require an appropriately sized arterial lumen and often a relatively healthy arterial wall) for hemostasis, ultrasound is intuitively important to a greater extent in TAVR. Ultrasound is applied to actively guide vessel puncture, with positioning often confirmed by fluoroscopy and femoral angiography. A systematic review of eight observational studies demonstrated that an ultrasound-guided approach, as compared to a fluoroscopic approach alone, was associated with a reduced risk of total, major, and minor access site complications [24]. Additionally, the Oxford TAVR registry found that ultrasound guidance for vascular access was associated with a significant reduction in a composite outcome of adverse events, driven by a reduction in vascular access complications [25]. While there are currently no randomized trials of ultrasound guidance for vascular access in TF TAVR, available nonrandomized evidence applicable to TAVR as well as the large body of evidence related to coronary angiography, coupled with the ease of use, availability, and lack of adverse effects from ultrasound, all make it an indispensable tool for percutaneous vascular access.

### 3.4. Vascular Closure

Effective hemostasis is the final step in safe transfemoral access for TAVR. Surgical cutdown facilitates direct repair of the arteriotomy; however, the specifics of this approach are beyond the scope of this review. For percutaneous access there are multiple vascular closure device (VCD) strategies that have developed as technology and expertise have evolved. These strategies rely on some combination of suture-based and plug-based devices [26].

#### 3.4.1. Commercial Devices

The main suture-based device is the Perclose Proglide (Abbott, Lake County, IL, USA) device, which allows for intravascular deployment of a pre-tied suture, which is subsequently tightened and locked to approximate an arteriotomy or venotomy. This is approved for sheaths up to 8 F when utilized after access has been obtained. It can also be used in sheaths up to 24 F when it is used in a “preclose” fashion. In this strategy, one or two Perclose devices are placed before insertion of the large-bore sheath without tightening of the knots. After sheath removal, these knots are tightened to approximate the arteriotomy. Importantly, the device allows for the maintenance of wire access after suture deployment, allowing for additional suture-based or plug-based devices if needed for effective hemostasis [26].

The alternative type of device used is the plug-based group of devices. These include the Angioseal (Terumo, Tokyo, Japan) and the Manta (Teleflex, Wayne, PA, USA). Both devices utilize opposed intravascular and extravascular components and are deployed at the completion of a procedure. The Angioseal comes in 6 F and 8 F sizes. It cannot be used solely for primary closure after TF TAVR but can be used in combination with a suture-based device, usually as a bailout strategy. Position within the vasculature is confirmed with blood flow through the device, at which point the device is deployed. The Manta device is a plug-based device that comes in two sizes, 14 F and 18 F. The device size is selected based on the vessel diameter, and both treat arteriotomies up to a 25 F outer diameter. It requires measurement of the vessel depth with a dedicated component of the device before large-bore access is obtained. After the procedure is completed, the device is placed over a wire to the predetermined depth and deployed. Both the Angioseal and the Manta then begin to undergo resorption. The Angioseal is fully reabsorbed at 90 days, while the Manta takes up to 6 months [26,27].

#### 3.4.2. Comparison of Closure Strategies

There are limited randomized trials of closure strategies comparing these devices. The MASH trial published by van Wiechen et al. randomized 210 patients undergoing TF TAVR with appropriate anatomy to a Manta device or two Proglide devices placed in a preclose fashion. The primary composite endpoint was access site-related major or minor complications at 30-day follow-up. Secondary endpoints included access site bleeding, time to hemostasis, and vascular closure device failure. There was no significant difference in the primary outcome. Vascular closure device failure was more common in the Proglide group, with the modes of failure distinct for the two devices [28].

The CHOICE-CLOSURE trial published by Abdel-Wahab et al. randomized 516 patients undergoing TF TAVR with appropriate anatomy to a Manta device or two Proglide devices with the option of an additional Angioseal if needed after 3 min of manual compression. The primary endpoint was access-site or access-related major and minor vascular complications. Secondary endpoints included rate of bleeding, vascular closure device failure, and time to hemostasis. The primary endpoint was significantly more common in the group randomized to Manta alone, with 19.4% in this group experiencing a complication as compared to 12% in the group randomized to the suture-based device strategy [29].

Lastly, the ACCESS-TAVI trial published by Rheude et al. randomized 454 patients undergoing TF TAVR to a closure strategy of one Proglide and one Angioseal or a strategy of two Proglides. The primary endpoint was a composite of major or minor access complications. Secondary endpoints included time to hemostasis, VARC bleeding grade 2 or worse, and all-cause mortality at 30 days. The two Proglide group had significantly higher rates of the primary outcome, driven by minor vascular complications, with the difference almost exclusively being the need for an additional device. In addition, time to hemostasis was longer and bleeding rates were higher in the two Proglide group [30].

Overall, there is varied data in these limited randomized trials. Notably, the rates of adverse events are higher than those reported in registry data, likely owing in part to the definitions used. However, this highlights that, at this time, provider experience and comfort with a particular strategy may be more important than the application of a specific strategy to all patients undergoing TF TAVR.

### 3.5. Complications

Vascular complications in TF TAVR are rare but can be devastating given the large size of the arteriotomy needed for the procedure. The Valve Academic Resource Consortium (VARC), founded in 2010, published its third set of endpoint definitions in 2021. The defined complication categories relevant to arterial access include bleeding complications, vascular access-related complications, and non-vascular access-related complications [31]. These are described in Table 1 and Table 2. Of note, completion angiography following the closure of large-bore vascular access can be performed to assess for evidence of either puncture site-related or non-puncture site-related complications. Some recommend this universally in large-bore access; however, in real-world practice, this is variable and may be performed only in individuals who are felt to be at high risk due to patient or procedural characteristics [32,33,34].

In the high-risk cohort undergoing TF TAVR with a 22 or 24 F arterial sheath in the PARTNER trial, at 30 days, 15.3% of patients had a major vascular complication, 11.9% had a minor vascular complication, and 11.3% had major bleeding. There was a significant association of major vascular complications with increased mortality [35]. Overall, the risk profile of patients undergoing TAVR has decreased [3]. At the same time, there have been improvements in anatomic assessment, device design, and operator experience, all leading to declining rates of access-related complications. A 2020 analysis of the STS/ACC TVT registry assessed patients from 2011 to 2016 and found that 9.3% of patients had any vascular complication while 7.6% had a bleeding event in-hospital [36]. These rates significantly decreased over time, from nearly 20% for both categories to levels near the overall rates reported. Additionally, both complications were associated with adverse outcomes, including death. An assessment of Medicare data of patients undergoing TAVR from 2012 to 2019 did not utilize VARC-3 definitions; nevertheless, it also demonstrated a continual reduction in rates of all complications, including hemorrhage [37]. Overall, access-related complications are variable in severity but, in aggregate, appear to be associated with adverse outcomes [38]. They are not common, and rates continue to decrease. Management strategy depends on the nature of the injury but typically relies on percutaneous or urgent surgical repair. Emphasis on pre-procedural planning and collaboration between interventional cardiology, cardiothoracic surgery, and vascular surgery is paramount to the safe execution of large-bore access and management of any ensuing complications [39].

### 3.6. Adjunct Procedures

Peripheral arterial disease (PAD) is highly prevalent both in TAVR trials and in registry data, likely owing to the age and comorbidities of this population [20]. Peripheral arterial disease can preclude the safe passage of the TAVR sheath. Endovascular techniques for managing PAD have been adopted to facilitate safe transfemoral access for TAVR, including balloon angioplasty and intravascular lithotripsy. Additional techniques, such as coating sheaths in a lubricious fluid such as propofol, have been described, but the variety of these is outside of the scope of this review [40]. The need and planning for these procedures should be anticipated based on the CT. Patients who require these procedures have higher periprocedural risk [41]. An assessment of patients undergoing TAVR in 2016 and 2017 identified in the Nationwide Readmissions database found that 4.4% of patients undergoing TAVR also had a peripheral intervention performed. These patients had higher mortality, stroke, acute kidney injury (AKI), need for blood transfusion, and longer hospital stays. However, when compared to patients undergoing alternate access TAVR, these patients who had a peripheral intervention to facilitate TF TAVR had lower mortality, AKI, median length of stay, and rates of 30-day readmission [42].

#### 3.6.1. Balloon Angioplasty

Angioplasty can be performed as the sole intervention or in combination with intravascular lithotripsy. In general, angioplasty allows for vessel expansion and plaque modification, which facilitates sheath insertion. Published experiences describe this as successful when applied to patients with small vessels and lesions that are not significantly calcified [43,44].

#### 3.6.2. Intravascular Lithotripsy

Intravascular lithotripsy is useful for the modification of heavily calcified peripheral arterial plaque. These stiff calcific lesions limit vessel distensibility and impede device delivery, and traditional balloon angioplasty may fail to modify them sufficiently. The Shockwave Balloon (Shockwave Medical, Santa Clara, CA, USA) is the only commercially available intravascular lithotripsy system. The balloons range from 2.5 mm to 12 mm in diameter and 40–110 mm in length. When inflated at nominal pressure at the level of a calcified lesion, pulses are applied through emitters along the balloon, which generate pressure waves through cavitation. These waves modify calcium, rendering diseased vessels more compliant. As it has become more widely adopted independently in peripheral and coronary applications, the utilization of this technology to enable TF TAVR has also increased, with a review of registry cases from 2018–2020 in six European centers showing an increase in its use from 2.4% to 6.5% of all procedures [45,46,47].

### 3.7. Femoral-Only TAVR Programs

Rates of non-TF TAVR are low, but most centers will develop expertise in one or more alternate access sites when femoral anatomy is unsuitable [3]. Given the large amount of experience with TF TAVR, the favorable ergonomics, commercially available closure devices, and improved outcomes associated with TF access, institutions may instead focus on the adjunct procedures described above to enable TF TAVR rather than utilize an alternate access site. Moccetti et al. published their institutional experience with one such protocol. From 2018 to 2021, their institution performed 400 TAVR procedures. Evaluation of the preprocedural CT allowed them to identify patients requiring balloon angioplasty or intravascular lithotripsy. Overall, only one patient was unable to undergo TF TAVR successfully due to aortic tortuosity. A small proportion (5.8%) of patients required a planned peripheral intervention, and an even smaller group (2%) required an unplanned intervention. A minority of patients (10.5%) had a minimal luminal diameter of less than 5.0 mm. These patients had more PAD, a higher degree of calcification, higher BMI, and lower ejection fraction. Compared to patients with a minimal luminal diameter greater than 5.0 mm, a larger proportion of patients (35.8%) required a peripheral intervention, and these patients had more bleeding and vascular complications. Overall, this cohort experienced similar rates of major complications and bleeding compared to contemporary registry data [48]. This suggests that careful preprocedural planning and appropriate vascular interventions can facilitate TF TAVR in most patients.

## 4. Alternative Access

As previously discussed, the vast majority of TAVR currently and historically have been performed via TF access. However, a percentage of patients will have unsuitable anatomy for TF TAVR and remain poor candidates for surgical aortic valve replacement, making alternative access routes important. Various sites have been developed since the introduction of TAVR; however, site preference has generally shifted from more invasive central access (transapical or transaortic) to less invasive peripheral access (transcarotid or transaxillary) (Figure 2) [3]. Landmark TAVR trials have largely included transfemoral access, with limited data on the safety profiles of alternative access routes. The utility of alternative access sites is largely informed by retrospective data [49]. Several procedural factors must be considered when considering alternative TAVR access, including the layout of the catheterization laboratory/operating room, procedure time, ergonomics, and radiation exposure. Institutional experience and learning curves play a significant role in reducing procedural times and radiation exposure. Proficiency with an alternative access modality seems achievable after 25–50 cases, although patient outcomes are similar before and after proficiency is achieved.

### 4.1. Transcarotid Access

#### 4.1.1. Indications and Patient Selection

Transcarotid (TC) TAVR requires common carotid arterial access for retrograde delivery of a TAVR prosthesis. This approach offers significant anatomical advantages for TAVR, particularly for patients with severely calcified or tortuous iliofemoral arteries, significant descending aortic pathology, or congenital aortic arch variants, where TF access may pose challenges. The carotid artery typically has less tortuosity and lower calcification, making it a suitable alternative [50,51]. TC access provides a direct route to the aortic valve, enhancing device control. Careful selection is essential, especially for patients with tortuous or heavily calcified carotid arteries, contralateral carotid stenosis > 50%, or congenital aortic arch variants. Prior carotid interventions, contralateral severe carotid stenosis or occlusion, and vertebral artery stenosis or occlusion are contraindications to this approach. Pre-TAVR imaging should assess carotid artery size (preferably > 6.5 mm) and vessel patency, with a CTA or MRI to confirm adequate collateral cerebral circulation [52].

#### 4.1.2. Procedural Technique

TC TAVR is usually performed in a hybrid operating room, typically requiring a surgical cutdown, although percutaneous access has been reported [53]. General anesthesia is the overwhelming preference, but conscious sedation may also be used. Of note, the use of general anesthesia has been associated with a higher rate of perioperative stroke, and special care must be taken for continuous monitoring of cerebral perfusion and maintenance of a higher target blood pressure to ensure cerebral blood flow [54]. The common carotid artery is exposed and clamped, and adequate distal perfusion is confirmed. A micropuncture needle and wire guide initial access, with a smaller sheath introduced before placing the main valve sheath to reduce occlusion risk. After valve deployment, the carotid artery is closed primarily [55]. A carotid angiogram should be performed after hemostasis to check for vessel patency and any vascular complications. Traditional catheterization laboratory setups do not facilitate the necessary positioning for safe TC access. This requires a change in room organization, which often places surgical operators close to radiation sources for fluoroscopy and interventional cardiologists near the level of the femoral artery or extending further cephalad. TC TAVR has been associated with significantly higher operator radiation exposure compared to the transfemoral TAVR, with a fourfold increase in radiation dose. While radiation exposure and procedure time are longer compared to TF procedures, it is shorter compared to other alternative access modalities.

#### 4.1.3. Outcomes and Complications

TC access shows comparable outcomes to other TAVR approaches, with similar stroke and 30-day mortality rates to TF and TAx access, as well as reduced atrial fibrillation, major bleeding, acute kidney injury, and shorter hospital stays compared to intrathoracic routes [56,57]. A prospective multicenter registry in France found a 30-day stroke rate of 1.6% in patients undergoing TC TAVR, a rate that is lower than in the PARTNER-2 trial. Direct comparison of these groups is not possible; however, their STS risk profile was similar, suggesting acceptable stroke risk in TC TAVR patients [58]. In obese patients, TC access has demonstrated lower vascular complication rates compared to transfemoral access, without significantly impacting mortality or major bleeding rates [59].

The TC approach offers improved device control and positioning due to its shorter, straighter access route, facilitating sheath delivery and valve implantation. This method enables earlier patient mobility, reduces hospital length of stay, and lowers the risk of immobility-related complications [60]. Additionally, bleeding risk is minimal and easier to manage, and the superficial access site allows for quicker hemostasis. Given the favorable outcomes and low complication rates, TC TAVR has become increasingly popular as an alternate access modality [61,62].

### 4.2. Transaxillary/Subclavian Access

#### 4.2.1. Indications and Patient Selection

Transaxillary/subclavian (TAx) TAVR requires axillary or subclavian artery access for retrograde delivery of the TAVR prosthesis. Patient selection requires pre-procedure CT imaging to assess the axillary and subclavian artery size (preferably larger than 6.5 mm), calcifications, tortuosity, and side branch locations [63]. Large-bore access should be avoided with an ipsilateral internal mammary graft to avoid the risk of dissection or occlusion during or after decannulation, jeopardizing coronary perfusion. The presence of an ipsilateral pacemaker or defibrillator may complicate access [64].

#### 4.2.2. Procedural Technique

In TAx TAVR, vascular access can be obtained surgically (with general anesthesia) or percutaneously (with general anesthesia or conscious sedation) [53,65]. The surgical approach involves an infraclavicular incision to expose the artery, which is then accessed with purse-string sutures. Alternatively, it can be obtained via a dacron conduit sewn onto the axillary artery, improving procedural ergonomics [66]. Percutaneous access, preferred for reducing pneumothorax risk, can be performed with imaging guidance [67]. The left axillary artery is generally chosen for its optimal coaxial alignment with the aortic annulus [68]. Contralateral radial access enables cerebral protection device placement, while femoral venous and arterial access can be used for ventricular pacing and aortogram, respectively. After valve deployment, hemostasis is obtained with vascular closure devices or primary surgical repair, depending on the access approach. An angiogram should be performed after hemostasis is achieved to verify vessel patency and assess for any vascular complications. TAx procedures are usually longer than TF due to the complexity of the access and imaging requirements. Operator radiation exposure is higher than TF TAVR but similar to TC TAVR. This is because the access location typically places operators near the fluoroscopic radiation source.

#### 4.2.3. Outcomes and Complications

Retrospective studies suggest TAx TAVR outcomes are comparable to TF TAVR, especially when using self-expanding valves. The Italian CoreValve Registry found similar success and complication rates (including major vascular complications, life-threatening bleeding, and 2-year mortality) between the approaches [69]. The reported rate of bail-out stenting and surgical vascular repair was found in one study to be 9.3% and 4%, respectively [70]. Compared to intrathoracic access, Cleveland Clinic data indicates that TAx TAVR results in fewer transfusions, shorter ventilation, less atrial fibrillation, and a shorter hospital stay, as well as similar stroke rates [71]. Ochsner Medical Center also reported faster extubation and shorter hospital stays for TAx TAVR than transapical TAVR [72].

### 4.3. Transapical Access

#### 4.3.1. Indications and Patient Selection

In contrast to the other access modalities, transapical (TA) TAVR requires surgical exposure of the left ventricle for myocardial access and antegrade delivery of the aortic valve prosthesis. This approach was the first widespread alternative access method. Due to procedural risks outlined below, transapical (TA) access is generally reserved for patients with no viable extrathoracic options and heavy ascending aortic calcification precluding transaortic access and is rarely needed in contemporary practice.

#### 4.3.2. Procedural Technique

TA access requires general anesthesia and involves a left mini-thoracotomy to expose the LV apex, which is then punctured [73]. Two purse-string sutures are placed, anticoagulation is administered, and a sheath is introduced over the wire into the left ventricle. The valve is deployed under rapid ventricular pacing with imaging guidance, and pacing is repeated during sheath removal and suture tying to reduce pressure until the repair is finished. Notably, this access is incompatible with self-expanding valves due to their delivery system, which does not allow antegrade delivery. The patient is positioned supine, with the left arm elevated. This can lead to ergonomic challenges for the operator, as well as increased radiation exposure when compared with TF access. The procedure is typically performed in a hybrid OR due to the need for surgical support. Additionally, a pleural drainage catheter is typically required after use of this access route. TA procedures are usually longer in duration due to the complexity of access.

#### 4.3.3. Outcomes and Complications

Observational studies suggest that TA TAVR is associated with worse outcomes than TF TAVR; however, this is confounded by the higher comorbidity burden in patients undergoing transapical access [74]. A meta-analysis of 2978 high-risk patients found increased 30-day mortality and major vascular events in the TA group compared to TF, though 1-year mortality was similar [75]. A propensity-matched analysis of the PARTNER I trial showed that patients who underwent TA access had more procedural complications, longer hospital stays, higher in-hospital and 6-month mortality, and lower aortic regurgitation incidence than TF patients [76]. The PARTNER II trial, which included intermediate-risk patients, found that TF TAVR was superior to SAVR, while transthoracic TAVR, including TA, showed no advantage over SAVR [77]. The STACCATO trial was terminated early due to excess adverse events in TA patients [78]. Additionally, analysis of the National Inpatient Sample from 2011–2017 revealed a sharp decline in TA TAVR use, from 27.7% in 2013 to 1.9% in 2017 [79]. This coincided with a decrease in peak mortality associated with TA TAVR from 5.53% in 2014 to 3.18% in 2017. However, it was still higher than the mortality from non-TA access, which was 1.24% in 2017.

TA TAVR carries distinct risks due to direct crossing of the LV apex, potentially causing myocardial stunning, necrosis, or a left ventricular aneurysm/pseudoaneurysm, all of which can impair ventricular function. Other complications may include damage to mitral valve chordae, leading to acute mitral insufficiency [74]. Furthermore, patients with chronic obstructive pulmonary disease often require prolonged postprocedural ventilation following TA TAVR compared to TF [80]. There is retrospective evidence, however, that at 30 days, both TA and TF access modalities are associated with favorable LV changes and AV hemodynamics, with similar 4-year survival, suggesting that if patients can safely undergo a TA TAVR, they can still receive similar long-term benefit [80].

### 4.4. Transaortic Access

#### 4.4.1. Indications and Patient Selection

Transaortic (TAo) TAVR relies on direct ascending aortic access for retrograde delivery of the aortic valve prosthesis. This access is reserved for patients ineligible for TF or other extrathoracic access. It is generally avoided in cases with thoracic deformities, very short or heavily calcified ascending aortas, or patent aorto-coronary grafts. Pre-TAVR CT imaging is essential to confirm at least 1 cm^2^ of calcium-free space for secure suture placement. Additionally, aortic trajectory should be assessed, as a horizontal aorta (angles over 70°) increases the risk of valve misalignment. The access point on the aorta should be positioned at least 6 cm above the aortic annulus to accommodate the delivery system [63].

#### 4.4.2. Procedural Technique

TAo access is achieved through a partial sternotomy or mini-thoracotomy, typically via the second, third, or fourth intercostal space, guided by preprocedural imaging [71]. This procedure requires general anesthesia and lacks a percutaneous option. After placing pledgeted purse-string sutures on the distal ascending aorta, needle access is obtained, followed by sequential dilation, sheath insertion, and valve deployment under rapid ventricular pacing and imaging. Upon completion, the purse-string sutures are tied under direct visualization. This approach involves a hybrid OR configuration with longer procedural times than the traditional TF approach. Radiation exposure is higher for TAo access than TF access but comparable to other alternative access modalities discussed above. The advantages of this method include cardiac surgeons’ familiarity with partial sternotomy and ascending aorta cannulation, the ability to quickly convert to full sternotomy if complications arise, reduced risk of myocardial damage and bleeding, and minimized chest wall injury [81,82].

#### 4.4.3. Outcomes and Complications

In high-risk patients, TAo TAVR may offer lower short- and long-term mortality than the transapical approach [74,83]. A retrospective study on 467 transfemoral, 42 transapical, and 289 transaortic cases (2011–2014) suggested a trend toward higher 1-year survival with transfemoral access, followed by transaortic, though the differences were not statistically significant [84]. In a propensity-matched study of 394 pairs, TAo TAVR patients had significantly higher rates of mortality, stroke, major bleeding, and acute kidney injury within 30 days compared to the transfemoral group [85]. A meta-analysis found similar 30-day mortality between TAo (7.9%) and TA (9.7%) groups, with a non-significant trend toward lower stroke rates in TAo access [86].

These findings suggest that, while TAo access may be favorable over TA access, extrathoracic alternatives are generally preferred when transfemoral access is unsuitable. The TAo approach has higher rates of serious bleeding when compared to TF TAVR, with 30-day bleeding rates reported as high as 66.7% [85]. A meta-analysis of 34 studies (32,689 patients) found that both TAo and TA access were associated with twice the 30-day mortality and 1.4 times the 1-year mortality risk compared to TF TAVR [87].

### 4.5. Transcaval Access

The first-in-human case series of 19 patients with TCv TAVR was reported by Greenbaum et al. in 2014, following preclinical animal studies by Lederman and colleagues in 2013 [12,88]. This approach provides alternative access for patients unsuitable for TF access due to small-caliber or severely diseased iliofemoral arteries. Through percutaneous femoral vein access, TCv entry into the abdominal aorta is achieved from the adjacent inferior vena cava by using electrosurgery techniques. Although this technique may seem counterintuitive, it is relatively safe because the retroperitoneal hydraulic pressure is higher than the venous pressure [89]. As a result, any arterial bleeding decompresses into the vena cava without harm as long as the venotomy is simultaneously unobstructed. This caval-aortic tract can be closed using a nitinol occluder device at the conclusion of the procedure. Although TCv access is described here in the context of alternative-access TAVR, this approach has also been described for the insertion of mechanical circulator support devices and treatment of aortic endoleaks.

Initially criticized, TCv access has gained acceptance with evidence of safety accumulated over the past decade. A propensity-weighted analysis found that it is associated with a fivefold lower risk of cerebrovascular accidents compared to TAx access (2.5% vs. 13.2%, OR: 0.20, 95% CI: 0.06–0.72; *p* = 0.014) without significant differences in major bleeding (10.0% vs. 13.2%) [90]. A meta-analysis further confirmed no significant differences in in-hospital or 30-day all-cause mortality, major bleeding, major vascular complications, or acute kidney injury between TCv and supra-aortic approaches, though neurovascular complications were numerically lower with TCv access (RR: 0.39, 95% CI: 0.14–1.09; *p* = 0.07) [90,91].

TCv access technique and procedure planning have been previously described comprehensively [89,92]. Here, we summarize the indications, planning, and step-by-step approach to TCv TAVR access and closure.

#### 4.5.1. Indication and Pre-Procedure Planning

TCv access is reserved for patients who are ineligible for femoral artery access, with case selection guided by a multidisciplinary heart team. The decision to undertake TCv access versus other alternative access routes discussed above will depend on patient anatomic factors and heart team expertise and preferences. Pre-procedure planning for TCv access involves a detailed analysis of a high-resolution (≤1 mm isotropic) contrast-enhanced CT of the abdomen and pelvis, ideally acquired during the same contrast exposure as the TAVR planning. Key objectives of the CT imaging include identifying an optimal target site, mapping lumbar vertebral and iliac artery landmarks for fluoroscopic and CT co-registration, defining fluoroscopic projection angles, and establishing bailout strategies, if necessary. The ideal target is a calcium-free window of at least 10 mm in one dimension facing the vena cava, free of interposed structures and at a safe distance from major branches to allow covered stent placement, if required. In case of significant bleeding requiring bailout balloon tamponade and covered stenting, the CT plan should specify the appropriate balloon diameter (typically, 120% of the aorta’s diameter at the traversal level) to achieve effective tamponade. It should also outline the diameter and length of a covered stent and confirm a suitable femoral artery access site, ensuring all necessary devices are prepared for rapid response. The femoral arterial access needed for bailout balloon and/or stenting devices needs to be prospectively planned and is a major limitation of TCv access, although rates for needing such bailout are quite low.

#### 4.5.2. Patient Preparation and Staff Briefing

TCv TAVR procedures can be performed under either general anesthesia or moderate sedation. Additional sedation or analgesia should be provided to awake patients just before electrosurgical traversal. Staff should be fully briefed on the procedural plan and prepared with all necessary equipment in case of severe bleeding requiring immediate intervention. Before draping, the electrosurgical dispersive electrode should be evenly applied to a clean, dry skin area (typically the thigh) to minimize skin burn risk and ensure effective electrosurgical function. The electrosurgery pencil should be connected to the monopolar terminal of the electrosurgery generator set to “pure” cutting mode at an initial power of 30 to 50 W.

#### 4.5.3. Transcaval Access Technique

**Figure 3:**
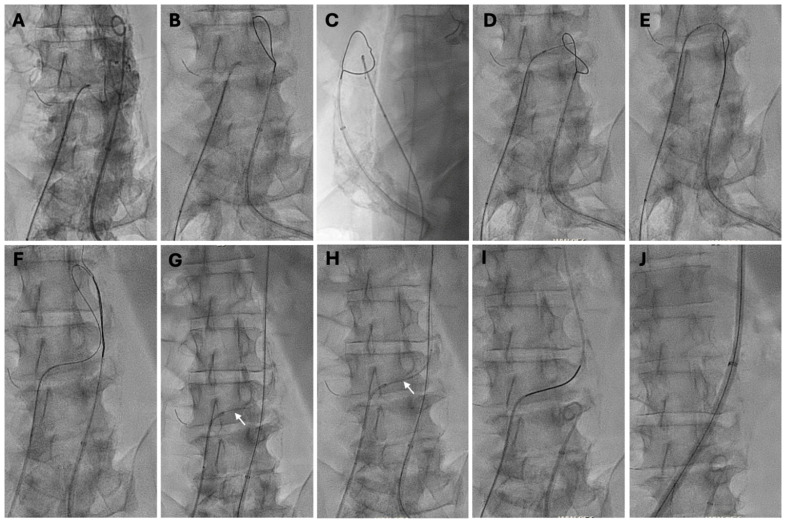
Transcaval Access (**A**) Traversal assembly at the site predetermined by computed tomography angle, and aortogram via the pigtail catheter. (**B**) Snare is positioned across the traversal system. (**C**) Position of the snare across the traversal system is confirmed in the orthogonal en face projection. (**D**) 0.014-inch guidewire is traversed across the vena cava and aorta into the snare with electrosurgery. (**E**) The aortic guidewire is ensnared while feeding forward to avoid outward traction on the aortic catheter. (**F**) The aortic guide is advanced, leading with a looped guidewire. (**G**) 0.014-inch microcatheter (arrow) is advanced over the 0.014-inch guidewire. (**H**) A 0.014-inch microcatheter (arrow) creates a 0.035-inch caval-aortic rail to deliver a 0.035-inch microcatheter. (**I**) A stiff 0.035-inch guidewire is delivered up to the aortic arch through the 0.035-inch microcatheter. (**J**) The transcatheter aortic valve replacement introducer sheath advances into the aorta.

The goal is to create a small entry point in the aortic wall to introduce a 0.014-inch guidewire, gradually enlarging the opening until the TCv TAVR introducer sheath can be positioned securely. Please refer to Figure 3 for sequential steps.

Step 1. Vascular access: Three vascular access sites (two venous and one arterial) are necessary. (1) The right femoral vein is preferred over the left to ease transaortic sheath advancement against resistance, and access should be obtained as cephalad as possible to reduce the skin-to-aorta distance when sheath length is limited. Suture-mediated closure devices (“preclosure”), “figure-of-8” suture, or a combination can be used as per local practice. (2) The larger femoral artery should be accessed to allow aortography, to position a snare target for transcaval guidewire traversal and ensnarement, and for possible bailout maneuvers, which may include delivery of an aortic tamponade balloon and/or covered stent implantation. (3) Venous access for temporary pacing for the TAVR valve implantation.

Step 2. Aortography and Venography: Position the pigtail aortography catheter below the renal arteries and align the transvenous guiding catheter near the traversal target, using a wide (~32 cm) fluoroscopic field of view. Rotate the C-arm to the CT-predicted frontal projection angle. Typically, a low-volume aortography (5–10 mL over 1 s) is sufficient. Many operators prefer digital subtraction for later comparison with post-closure aortograms, while unsubtracted images are useful for showing bony landmarks during traversal. Baseline or completion vena cava angiography is unnecessary and adds little to the procedure. 

Step 3. Insertion of Snare and Venous Crossing System: After administering the full dose of heparin to achieve the target ACT > 250 s, the crossing assembly system from the right femoral vein is positioned based on the CT-derived angles. This co-axial system comprises a rigid, exchangeable 0.014-inch × 300 cm guidewire (e.g., Astato XS 20 or amputated Confianza Pro 12, Asahi, Irvine, CA, USA) housed within a 0.014-inch microcatheter (preferably the 145-cm PiggyBack wire converter, Teleflex, Wayne, PA, USA). This is further enclosed within a 0.035-inch braided microcatheter, ideally with a lubricious coating (e.g., 0.035-inch 90-cm NaviCross, Terumo, Tokyo, Japan), which is connected to a rotating hemostatic adapter on a 6- to 8-F guiding catheter (such as a 55-cm internal mammary, short renal double curve 1, or deflectable guiding sheath). A single-loop snare (e.g., Amplatz Goose Neck, Medtronic, Dublin, Ireland) with a diameter 5–10 mm larger than the aorta is loaded into a 6 Fr guiding catheter (like JR4). The snare is positioned cephalad to the target and gradually withdrawn while applying torque until it rests along the right aortic wall at the target level.

Step 4. Electrosurgical Crossing: The insulation on the back end 1 cm of the 0.014-inch guidewire is stripped using a scalpel to enable electrical conduction and clamped to the electrosurgical pencil with metallic forceps. The traversal guiding catheter is precisely positioned toward the target by alternating between frontal and orthogonal en face projections as determined by the CT, torquing to align with the snare “bull’s-eye” in the lateral en face view. The cutting mode, typically activated by the yellow switch on electrosurgical pencils, should be used deliberately and briefly. The electrified guidewire should be advanced immediately at a steady pace of approximately 2 mm/s for no more than 2–3 s per activation. Electrification must be stopped as soon as the guidewire enters the aorta, confirmed by angiography and the prepositioned snare. Once the guidewire reaches the aorta, it should be advanced further without additional electrification until slight buckling is observed against the contralateral aortic luminal wall. 

Step 5. Countertraction and exchange for a rigid 0.035-inch guidewire: The guidewire should be carefully ensnared and advanced, ensuring minimal traction on the ensnared guidewire. Excessive pulling should be avoided by advancing redundant guidewire into the aorta from the IVC while simultaneously torque-advancing the aortic guiding catheter. The guidewire is then invaginated into the aortic guiding catheter. The transfemoral aortic guiding catheter is advanced to the level of the aortic arch, with the transcaval guidewire fed antegrade. Excessive traction can cause the aortic guiding catheter to be pulled through the aortotomy, causing injury, and should be avoided by feeding excess wire from the IVC. Countertraction between the caval and aortic guiding catheters straightens the guidewire, allowing the 0.014-inch microcatheter to advance across the transcaval tract into the thoracic aorta. Subsequently, the 0.035-inch microcatheter is advanced over the 0.014-inch microcatheter deeply into the aorta to the level of the thoracic descending aorta. The snare is then released from the 0.014-inch guidewire. The 0.014-inch guidewire and 0.014-inch microcatheter are withdrawn. A rigid 0.035-inch guidewire (e.g., single-curve Lunderquist Extra-Stiff, Cook Medical, Bloomington, IN, USA) is advanced through the 0.035-inch microcatheter under fluoroscopic guidance, ensuring the microcatheter remains in the aorta without prolapsing into the cava or retroperitoneal space, and positioned just distal to the aortic arch. The 0.035-inch microcatheter, IVC guiding catheter, and femoral venous sheath are removed, and the TAVR introducer sheath is then advanced into the aorta under fluoroscopic guidance, ensuring the sheath tip does not “flare” or cause injury to the aortic wall. Confirm that the sheath has secure purchase within the aorta before proceeding. The TAVR procedure is subsequently performed following standard steps. 

#### 4.5.4. Closure

**Figure 4:**
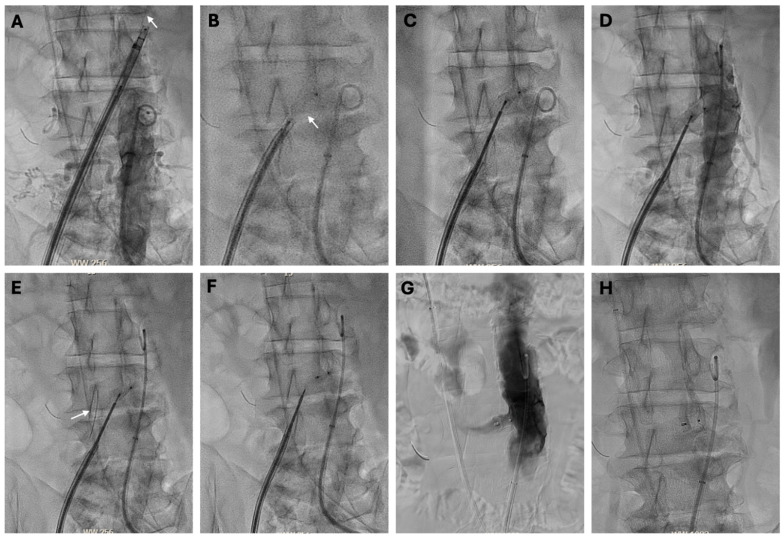
Transcaval Closure (**A**) A 0.014-inch “buddy guidewire” (arrow) across the aortocaval tract allows recrossing in case of inadvertent device pull-through. (**B**) Partially exposed (“bulbed”) nitinol occluder (arrow). (**C**) “bulbed” occluder turns sideways when withdrawn while deflecting the guiding sheath. (**D**) Aortogram before releasing the nitinol occlude for the confirmation of the position. (**E**) 0.014-inch “buddy guidewire” (arrow) is withdrawn back into the vena cava after the device position is confirmed and before releasing the device. (**F**) The nitinol occluder is released. (**G**) Completion aortogram shows the “funnel”-shaped aortocaval fistula, which usually resolves within a few days to weeks. (**H**) Completion cine image of the occluder device.

After reversing heparin with intravenous protamine, closure is performed using a nitinol occluder, preferably the first-generation Amplatzer Duct Occluder (ADO-1, size 10/8, model 9-PDA-006, Abbott Vascular), featuring a 16 mm aortic disc (“retention skirt”) and an 8–10 mm unconstrained neck diameter. The TCv sheath is replaced with a deflectable guiding sheath for sideways deployment. An 0.014-inch 300 cm guidewire placed alongside the deflectable sheath maintains transcaval access should the closure device “pull through” the aortotomy during device formation; this wire is retracted once the device is placed with satisfactory closure. The sequential steps of the closure are outlined in Figure 4. The goal is not immediate occlusion of the aortic hole but rather the creation of a manageable aortocaval fistula that closes over minutes to weeks. Digital subtraction aortography is performed in the updated projection angle, typically using 10–15 mL of contrast injected over 1 s during a breath hold, with prolonged imaging (5–10 s) to detect subtle extravasation. Troubleshooting related to this access is beyond the scope of this review and have been described previously [86].

#### 4.5.5. Complications

TCv access for TAVR is associated with higher rates of bleeding and vascular complications compared to transfemoral and transcarotid routes, with bleeding complications similar to the transaxillary approach [34,90,92,93,94,95]. Life-threatening bleeding occurs in approximately 7% of cases, major vascular complications in 13%, blood transfusions are required in 23.3%, and retroperitoneal bleeding is reported in 19% [96,97]. Despite these relatively higher rates of bleeding complications, bailout covered stents are required in only 1% of cases [96]. Residual caval-aortic fistulae typically close spontaneously within hours to months in almost all cases, with only 1% remaining patent at 12 months, and no subacute or late complications such as vascular issues, occluder failure, or migration have been reported [89].

### 4.6. Selection of Access Site

As described above, there are various anatomic and procedural considerations that influence the selection of access site for performance of TAVR. These are briefly described above (Figure 1) and also more comprehensively below (Table 3). While there have been no randomized trials comparing access sites, the authors feel that the information discussed above can lead to a practical approach for site selection. First, TF TAVR is preferred for many reasons including ease of use, ability for percutaneous approach and moderate sedation, low rate of complications, and the strength of the randomized TAVR data which relied on this route. If TF TAVR is not feasible, an alternate extrathoracic route should be considered, which includes TC, TCv, and TAx. The selection among these sites should be dependent primarily on appropriate anatomy and institutional experience. If these sites are not suitable, an intrathoracic route can be considered, which includes TAo and TA. Selection of an intrathoracic route should carefully consider the complexity of surgical exposure, the associated unique complications, and increased recovery time. Surgical AVR should be considered if there is significant concern about the ability to perform TAVR related to either vascular access or valve implantation; however, in a patient with sufficient complexity to raise concerns regarding TAVR, it is very likely that there will be a similar level of concern with performing SAVR.

## 5. Future Directions

Since its inception in 2002, TAVR is safer, and complication rates have decreased. This is due to increased provider experience as well as the development of improved technology and techniques. This innovation continues both related to the valve prosthesis with emphasis on procedural outcomes such as minimizing conduction disturbances and paravalvular regurgitation and related to the vascular access needed for the procedure. The Xemed valve (Medinol, Tel Aviv, Israel) is a modular device that, due to its unique design, can be performed with a smaller arteriotomy. The system requires a 12 F sheath for access. There are two modules of the system: an anchor module, which is deployed first, and a valve module, which is assembled in the aorta and attached to the anchor to form a functioning prosthesis. The first-in-human case was presented in 2023, in an 88-year-old patient with high surgical risk and severe peripheral arterial disease requiring cutting balloon angioplasty for placement of the delivery sheath. Post-implant peak-to-peak gradient was measured at four mmHg. The company plans to further reduce the size of the system to 9 F [115]. In addition to refinement of the primary access site, emphasis on the secondary arterial and venous access for TAVR (typically for angiography and rapid ventricular pacing, respectively) may help improve patient outcomes. A study published by Versteeg et al. randomized patients to an upper-extremity or lower-extremity approach for secondary arterial and venous access. The upper-extremity group had radial artery and upper arm vein access obtained, while the lower-extremity group had femoral artery and vein access obtained. The primary outcome of bleeding was significantly higher in the lower-extremity group (13.4% vs. 4.2%); however, the upper-extremity procedure was longer on average (60 vs. 48 min) [116]. As throughout the development of TAVR, advancements to improve patient outcomes will rely both on technological innovations as well as the creative application of these technologies in clinical practice. 

## 6. Conclusions

TAVR is an integral tool in the management of aortic stenosis, with its success fueling efforts into the development of transcatheter tools for the management of other forms of valvular heart disease. Its dissemination has been supported by the ability to perform the procedure safely and in variant anatomies. As the above review describes, methods are available for vascular access to enable valve delivery in nearly all anatomies. Outcomes of all access methods have improved with time and should continue to improve with further development of devices and procedural techniques. Transfemoral access remains the site of choice in appropriate anatomy. The decision to use advanced endovascular techniques to enable TF access or the decision to pursue an alternate access site should be made through a collaborative heart team approach based on patient characteristics and operator experience.

## Figures and Tables

**Table 1:** Valve Academic Research Consortium—Bleeding and transfusion *.

Types	Definition Overt Bleeding ^†^ That Fulfils One of the Following Criteria:
Type 1 Minor	Overt bleeding that does not require surgical or percutaneous intervention, but does require medical intervention by a health care professional, leading to hospitalization, an increased level of care, or medical evaluation (BARC 2) Overt bleeding that requires a transfusion of one unit of whole blood/red blood cells (BARC 3a) ^‡^
Type 2 Major	Overt bleeding that requires a transfusion of two to four units of whole blood/red blood cells ^‡^ (BARC 3a) Overt bleeding associated with a haemoglobin drop of >3 g/dL (>1.86 mmol/L) but <5 g/d (<3.1 mmol/L) (BARC 3a)
Type 3 Life threatening	Overt bleeding in a critical organ, such as intracranial, intraspinal, intraocular, pericardial (associated with haemodynamic compromise/tamponade and necessitating intervention), or intramuscular with compartment syndrome (BARC 3b, BARC 3c) Overt bleeding causing hypovolemic shock or severe hypotension (systolic blood pressure < 90 mmHg lasting > 30 min and not responding to volume resuscitation) or requiring vasopressors or surgery (BARC 3b) Overt bleeding requiring reoperation, surgical exploration, or re-intervention for the purpose of controlling bleeding (BARC 3b, BARC 4) Post-thoracotomy chest tube output ≥ 2 L within a 24-h period (BARC 4) Overt bleeding requiring a transfusion of ≥5 units of whole blood/red blood cells (BARC 3a) ^‡^ Overt bleeding associated with a haemoglobin drop ≥5 g/dL (≥3.1 mmol/L) (BARC 3b)
Type 4 Leading to death	Overt bleeding leading to death. Should be classified as: Probable: Clinical suspicion (BARC 5a) Definite: Confirmed by autopsy or imaging (BARC 5b)

* The timing, indication, and number of transfused blood products should be collected and reported specifically during the index procedure, during the entire index hospitalization, and during follow-up after discharge, whether or not overt bleeding is identified. ^†^ Overt bleeding is defined as any clinically obvious source of bleeding or bleeding source identified after appropriate investigation and diagnostic testing (e.g., imaging). Any procedural blood loss should be considered overt bleeding. ^‡^ Total number of transfusions should be reported separately for (i) within 48 h of the index procedure, (ii) the total duration of the index procedure hospitalization, and (iii) during any subsequent repeat hospitalization.

**Table 2:** Vascular and access-related complications *.

Type	Criteria	Definition
Access^-^related Vascular ^†^	Major	One of the following: Aortic dissection or aortic rupture Vascular (arterial or venous) injury (perforation, rupture, dissection, stenosis, ischaemia, arterial or venous thrombosis, including pulmonary embolism, arteriovenous fistula, pseudoaneurysm, haematoma, retroperitoneal haematoma, infection) or compartment syndrome resulting in death, VARC type ≥ 2 bleeding, limb or visceral ischaemia, or irreversible neurologic impairment Distal embolization (non-cerebral) from a vascular source resulting in death, amputation, limb or visceral ischaemia, or irreversible end-organ damage Unplanned endovascular or surgical intervention resulting in death, VARC type ≥ 2 bleeding, limb or visceral ischaemia, or irreversible neurologic impairment Closure device failure ^‡^ resulting in death, VARC type ≥ 2 bleeding, limb or visceral ischaemia, or irreversible neurologic impairment
	Minor	One of the following: Vascular (arterial or venous) injury (perforation, rupture, dissection, stenosis, ischaemia, arterial or venous thrombosis, including pulmonary embolism, arteriovenous fistula, pseudoaneurysm, haematoma, retroperitoneal haematoma, infection) not resulting in death, VARC type ≥ 2 bleeding, limb or visceral ischaemia, or irreversible neurologic impairment Distal embolization treated with embolectomy and/or thrombectomy, not resulting in death, amputation, limb or visceral ischaemia, or irreversible end-organ damage Any unplanned endovascular or surgical intervention, ultrasound-guided compression, or thrombin injection, not resulting in death, VARC type ≥ 2 bleeding, limb or visceral ischaemia, or irreversible neurologic impairment Closure device failure ^‡^ not resulting in death, VARC type ≥ 2 bleeding, limb or visceral ischaemia, or irreversible neurologic impairment
Access related non-vascular	Major	One of the following: Non-vascular structure, non-cardiac structure ^§^ perforation, injury, or infection resulting in death, VARC type ≥ 2 bleeding, irreversible nerve injury, or requiring unplanned surgery or percutaneous intervention Non-vascular access site (e.g., trans-apical left ventricular) perforation, injury, or infection resulting in death, VARC type ≥ 2 bleeding, irreversible nerve injury, or requiring unplanned surgery or percutaneous intervention
	Minor	One of the following: Non-vascular structure, non-cardiac structure ^§^ perforation, injury, or infection not resulting in death, VARC type ≥ 2, irreversible nerve injury, or requiring unplanned surgery or percutaneous intervention Non-vascular access site (e.g., trans-apical left ventricular) perforation, injury, or infection not resulting in death, VARC type ≥ 2 bleeding, irreversible nerve injury, or requiring unplanned surgery or percutaneous intervention

* Any complication related to the device insertion, delivery, and complete removal of all its components (delivery catheter, sheath, guide wire), excluding the actual implantation in the heart. ^†^ Any device-related vascular access site and any other accessory access sites (venous or arterial) used during the procedure. ^‡^ A failure to achieve haemostasis at the access site, resulting in alternative treatment (other than manual compression or planned adjunctive endovascular balloon inflation). ^§^ Including, but not limited to, the lung (e.g., pneumothorax), direct nerve injury, access site or wound infection, mediastinitis, sternal instability, wound dehiscence, and inability to close the chest.

**Table 3:** Access-site considerations.

Access Route	Usage (%) [3]	Indications	Contraindications	Major Complications
Transfemoral	95.26%	Most patients with adequate iliofemoral anatomy Minimum vessel diameter 5.0–6.0 mm	Severe iliofemoral disease, small vessel diameter (<5 mm), heavy calcification, severe tortuosity Aortic dissection or aneurysms, congenital variants Prior vascular interventions Unfavorable body habitus or extreme obesity	Vascular complications: up to 15% Major bleeding: 11.3% New LBBB: 14.8% PPI: 7.9% Stroke: 3.8% Intrathoracic complications (2.9%): pericardial effusion, cardiac perforation, valve dislodgement. Endocarditis: 1% [98,99,100]
Transcarotid	0.91%	Alternative access when transfemoral TAVR is contraindicated Adequate carotid artery size (>6.5 mm) and vessel patency, confirmed via imaging	Severe carotid artery disease (including significant contralateral stenosis) Prior carotid interventions History of stroke Anatomical concerns precluding cerebral embolic protection device placement	Major bleeding: 7% Vascular complications: 2.5% PPI: 2.1–16.7 Stroke: 3.9% Tamponade: 1.7% [56,94,101,102]
Transaxillary Transsubclavian	2.49%	Alternative access when transfemoral TAVR is contraindicated Axillary or subclavian artery size > 6.5 mm	Severe subclavian artery disease, history of chest radiotherapy Ipsilateral internal mammary artery graft Ipsilateral pacemaker or defibrillator	Vascular complications: up to 9.3% PPI: 3.6–28.5% Stroke: 8–13% [70,71,72,90,103,104,105]
Transapical	0.29%	Last-resort access for patients without viable extrathoracic options	Severe left ventricular dysfunction Severe apical calcification Prior LV surgery Incompatibility with self-expanding valves	Mortality: up to 10% Vascular complications: 2.4% PPI: 5.9–20.5% Stroke: 0.6–3.1% Mechanical complications: direct myocardial injury, left ventricular aneurysm/pseudoaneurysm, mitral valve damage, tamponade Endocarditis: 1–1.4% [106,107,108,109,110]
Transaortic	0.47%	Alternative access when transfemoral TAVR is contraindicated At least 1 cm^2^ of calcium free space for secure suture placement Adequate distance (≥6 cm) from the aortic annulus to accommodate the delivery system	Thoracic deformities or very short ascending aorta Severely calcified ascending aorta Patent aorto-coronary bypass grafts Severe horizontal aorta (≥70° angle)	Mortality: 9.9% Major bleeding: up to 66.7% Vascular complications: 3.1% PPI: 7.1–11.7 Stroke: 3.7% [111,112,113,114]
Transcaval	0.17%	Alternative access when transfemoral TAVR is contraindicated Optimal calcium-free window (≥10 mm) in the abdominal aorta, adjacent to the inferior vena cava, safe distance from major arterial branches	Severe aortic calcification or aorto-iliac disease Interposed anatomical structures No suitable femoral arterial access for emergency bailout maneuvers	Mortality: 6.1% Bleeding: 7–23.3% Vascular complications: 13% Caval-aortic persistent fistulae: 1% Stroke: 3.3% [93]

LBBB: Left Bundle Branch Block, PPI: Permanent Pacemaker Implantation.

## Data Availability

No new data were created or analyzed in this study. Data sharing is not applicable to this article.
